# Inflammatory Biomarkers and Carotid Atherosclerosis: The Predictive Role of the Neutrophil/Albumin Ratio

**DOI:** 10.3390/medicina61081495

**Published:** 2025-08-21

**Authors:** Halis Yilmaz, Cemre Turgul, Yucel Yilmaz, Saban Kelesoglu, Aydin Tuncay

**Affiliations:** 1 Department of Cardiovascular Surgery, Erciyes University Faculty of Medicine, 38039 Kayseri, Türkiye; halisy38@hotmail.com; 2 Department of Cardiology, University of Health Sciences, Kayseri City Training and Research Hospital, 38080 Kayseri, Türkiye; cemrebarhana@hotmail.com (C.T.); dryyilmaz@hotmail.com (Y.Y.); 3 Department of Cardiology, Erciyes University Faculty of Medicine, 38039 Kayseri, Türkiye; dr.s.k@hotmail.com

**Keywords:** critical carotid artery stenosis, neutrophil/albumin ratio, atherosclerosis, biomarker

## Abstract

*Background and Objectives*: Carotid artery stenosis is an inflammatory vascular disease closely linked to atherosclerosis and associated with inflammatory biomarkers. The neutrophil/albumin ratio (NAR) is a novel promising biomarker in assessing cardiovascular disease severity. This study aimed to evaluate the relationship between NAR and lesion severity in patients with carotid artery stenosis. *Materials and Methods*: This retrospective, single-center, comparative study included 625 asymptomatic patients who underwent digital subtraction angiography (DSA) for suspected high-grade carotid artery stenosis between 2012 and 2022. Patients were classified into two groups based on stenosis severity: critical carotid artery stenosis (≥70% stenosis) and non-critical carotid artery stenosis (<70%). Only asymptomatic patients were included; patients with symptoms were excluded. NAR was calculated preoperatively as neutrophil count divided by serum albumin. Additional inflammatory markers, such as neutrophil–lymphocyte ratio (NLR) and C-reactive protein (CRP) to albumin ratio (CAR), were also analyzed. *Results*: Severe carotid artery stenosis was detected in 191 of the patients who underwent DSA. Individuals in the critical carotid artery stenosis group were older and had a higher prevalence of diabetes mellitus and hypertension (51 (45–57) vs. 60 (54–68), *p* < 0.001; 143 vs. 83, *p* = 0.025; 193 vs. 104, respectively, *p* = 0.021), as well as higher neutrophil counts (4.3 (3.2–6.2) vs. 8.1 (4.9–12.5), *p* < 0.001), NLR (2.2 (1.4–3.2) vs. 4.2 (2.3–8.9), *p* < 0.001), while CRP (3.8 (1.8–8) vs. 5.7 (3.6–7.6), *p* = 0.005) and CAR (0.9 (0.5–1.9) vs. 1.6 (0.8–2.1), *p* < 0.001) values were significantly higher. NAR was higher in patients of the critical carotid artery stenosis group than the non-critical (1.1 (0.8–1.6) vs. 2.1 (1.4–3.2), *p* < 0.001). Multivariate analysis identified NAR as an independent predictor of carotid artery stenosis (Odds Ratio [OR]: 3.432; 95% Confidence Interval [CI]: 2.116–5.566; *p* < 0.001). The best cut-off value of NAR for predicting critical carotid artery stenosis was 1.47, which provided 73.8% sensitivity and 70.5% specificity. *Conclusions*: NAR, which can be easily measured through a simple blood test, demonstrated moderate sensitivity and specificity in predicting critical carotid artery stenosis, suggesting its potential role as a supportive marker in clinical risk assessment.

## 1. Introduction

Atherosclerotic cardiovascular diseases (CVD) are still among the most important health problems in terms of mortality and disability-adjusted life years (DALY) worldwide. According to recent data, the global burden of DALYs attributed to CVD is approximately twice the amount of DALYs due to neoplasms [1]. Carotid artery stenosis, characterized by the accumulation of atherosclerotic plaque in the carotid arteries, generally affects approximately 5–7% of individuals over 50 years of age [2,3]. More than 80% of all strokes are ischaemic in origin, and carotid artery stenosis is responsible for approximately 20% of these strokes [3,4,5]. The presence of atherosclerotic plaques in the carotid arteries and the assessment of plaque burden play an important role in the prediction of cerebrovascular event risk [6,7,8,9].

Severe carotid artery stenosis significantly increases the risk of ischaemic stroke, primarily due to embolization from unstable plaques. Although less frequent, impaired cerebral perfusion may contribute, particularly in carotid artery stenosis with poor collateral circulation or bilateral stenosis [10]. Progression and/or rupture of plaques leads to thrombotic, occlusive, or embolic events, and this process is closely associated with inflammatory biomarkers, whose quantification provides important information [11,12]. However, the multifactorial nature of CVD complicates factors determination. Although current treatment and follow-up strategies have significantly improved the clinical outcomes, adverse events such as plaque progression, cerebrovascular incidents, or restenosis may occur unpredictably in certain patients. This unpredictability underscores the need for additional risk stratification tools, including inflammatory biomarkers, and the development of personalized diagnosis and treatment strategies [13]. Many inflammatory biomarkers are frequently preferred due to their low-cost, easily applicable, reproducible, non-invasive nature [14,15,16]. A new marker called neutrophil/albumin ratio (NAR) was initially evaluated in oncological diseases, but today it has started to be the subject of research in different clinical entities such as CVD [17,18,19,20,21].

While duplex ultrasound (DUS) remains the cornerstone imaging modality for assessing carotid plaque morphology and stenosis severity—owing to its non-invasive nature, wide availability, low cost, and lack of radiation exposure—its diagnostic accuracy can be influenced by operator experience and patient-specific anatomical factors. In this context, the integration of additional inflammatory biomarkers may provide complementary information, particularly in early disease stages or in patients with ambiguous imaging findings [22,23,24,25]. These markers may aid in refining risk stratification, optimizing surveillance intervals and enhancing medical management strategies. However, there is no study in the literature that specifically evaluates the relationship between NAR and carotid artery stenosis. This study aimed to investigate the association between lesion severity and the NAR in patients with asymptomatic carotid artery stenosis and to assess its potential role as a supportive tool in clinical decision-making.

## 2. Materials and Methods

This retrospective, single-center study included 625 asymptomatic patients who underwent carotid DUS for various reasons between 2012 and 2021 and had carotid artery stenosis of 70% or more at the time of diagnosis. Initial screening and surveillance were performed using DUS, and patients with suspected high-grade stenosis (≥70%) underwent confirmation of diagnosis by digital subtraction angiography (DSA). In this protocol, DSA served both as a diagnostic confirmation tool and as a pre-intervention step to guide therapeutic decisions such as carotid endarterectomy or stenting. Patients with stenosis below 70% were not included in the study unless they had progressed to ≥70% at the time of diagnosis. For the purpose of statistical analysis and correlation with symptoms and imaging findings, we counted each carotid artery separately as an individual data point. In patients with bilateral carotid stenosis, we selected the more severely stenotic side for detailed analysis, especially when comparing imaging findings with clinical presentation. Those with stable stenosis below this threshold were managed conservatively with regular clinical and DUS follow-up and appropriate medical treatment.

In our institution, DSA is routinely performed as a confirmatory imaging modality in patients with ≥70% carotid stenosis on duplex ultrasound who are being considered for carotid artery stenting. This practice is based on clinical protocols that prioritize precise lesion characterization and procedural planning, as well as the ability to proceed with treatment in the same session. DSA is particularly valuable in our setting due to limited access to CTA and MRA in public hospitals, long waiting times, and reimbursement restrictions imposed by the national health system. It also avoids potential risks associated with CTA-induced nephropathy and is suitable for patients who have contraindications to MRA, such as claustrophobia or metallic implants.

To avoid confounding effects on baseline inflammatory and biochemical markers, particularly neutrophil and albumin levels, which are components of NAR, the following exclusion criteria were applied: acute stroke (ischaemic or haemorrhagic), severe head trauma, bilateral severe carotid artery stenosis on DUS and/or DSA (≥70%), previous coronary artery bypass graft surgery (CABG), acute coronary syndrome or percutaneous coronary intervention within the last 3 months, heart failure, hematologic diseases, malignancy, active infection or systemic inflammatory diseases, use of anti-inflammatory medications, and severe renal or hepatic insufficiency.

All patients underwent transthoracic echocardiography prior to carotid angiography to assess cardiac function. Left ventricular ejection fraction (LVEF) was calculated using the Simpson method. The study protocol was approved by the local ethics committee in accordance with the Declaration of Helsinki (Ethics Committee Approval: 2022/117).

### 2.1. Laboratory Analyses

Antecubital venous blood samples were collected on an empty stomach in the morning before DSA, and comprehensive biochemical and complete blood count analyses were performed. Biochemical tests were performed with COBAS^®^ c701 (Roche Diagnostics, Mannheim, Germany) automated analyzer, and hematological parameters were evaluated with Sysmex K-1000 Haematology Analyzer (Cobe, Japan) within 30 min after sampling. NLR was calculated by dividing the number of neutrophils by the number of lymphocytes; CAR was calculated by dividing the C-reactive protein (CRP)/albumin ratio (CAR) level by the albumin level; and NAR was calculated by dividing the number of neutrophils by the albumin level.

### 2.2. Digital Subtraction Angiography (DSA)

DSA was performed by experienced interventional cardiologists using the conventional Seldinger technique via the femoral artery access. A non-ionic, low-osmolar contrast agent (Iohexol, Omnipaque 350 mg/mL; GE Healthcare, Cork, Ireland) was used in all procedures. The severity of carotid artery stenosis was assessed according to the North American Symptomatic Carotid Endarterectomy Trial (NASCET) criteria, and the percentage of stenosis was calculated using electronic calipers on the Picture Archiving and Communication System (PACS) [26]. Both lateral and anteroposterior projections were evaluated, and measurements were taken at the narrowest point of the lumen. Stenosis of 70% or greater was classified as critical carotid artery stenosis, while stenosis below 70% was considered non-critical.

### 2.3. Statistical Analysis

Statistical analyses were performed using SPSS 23.0 (SPSS Inc., Armonk, NY, USA). The normality of distribution of the variables was evaluated by the Shapiro-Wilk test; data showing normality were presented as mean ± standard deviation, and those not showing normality were presented as median and interquartile range (IQR). The Mann-Whitney U test was used to compare non-normally distributed data, and the independent samples t-test was used for normally distributed data. Factors affecting the occurrence of critical carotid artery stenosis were determined by univariate analysis, and parameters with *p* < 0.1 were included in the multivariate regression model. ROC (Receiver Operating Characteristic) curve analyses were performed for NAR and NLR to predict critical carotid artery stenosis, and ideal cut-off values were selected as thresholds providing maximum sensitivity and specificity. In all analyses, a two-way *p* < 0.05 was accepted as the limit of statistical significance.

## 3. Results

This study included patients (*n* = 625) who underwent DSA for carotid artery stenosis assessment. Patients were divided into two groups as critical and non-critical carotid artery stenosis. Demographic and clinical characteristics of the groups included in the study are presented in Table 1. When both groups were compared, it was determined that the age of the individuals in the critical carotid artery stenosis group was higher, and the prevalence of diabetes mellitus (DM) and hypertension (HT) was significantly higher in the critical carotid artery stenosis group (51 (45–57) vs. 60 (54–68), *p* < 0.001; 143 vs. 83, *p* = 0.025; 193 vs. 104, *p* = 0.021, respectively).

When compared in terms of laboratory findings, neutrophil count (4.3 (3.2–6.2) vs. 8.1 (4.9–12.5), *p* < 0.001), NLR (2.2 (1.4–3.2) vs. 4.2 (2.3–8.9), *p* < 0.001), CRP (3.8 (1.8–8) vs. 5.7 (3.6–7.6), *p* = 0.005) and CAR (0.9 (0.5–1.9) vs. 1.6 (0.8–2.1), *p* < 0.001) values were significantly higher. When the two groups were compared in terms of NAR, it was observed that NAR values were significantly higher in patients in the critical carotid artery stenosis group (1.1 (0.8–1.6) vs. 2.1 (1.4–3.2), *p* < 0.001) (Table 2).

The results of multivariate analyses performed to evaluate different risk factors affecting carotid artery stenosis severity are presented in Table 3. Age, HT, DM, white blood cell (WBC), NLR, CRP, CAR, and NAR values, which were found to be associated with carotid artery stenosis severity in the univariate analysis, were included in the multivariate logistic regression analysis. As a result of the analysis, high NAR was found to be an independent predictor of critical carotid artery stenosis (Odds Ratio [OR]: 3.432; 95% Confidence Interval [CI]: 2.116–5.566; *p* < 0.001). In addition, advanced age, presence of HT, and high NLR values were also found to be independent predictors of critical carotid artery stenosis (OR: 1.120, 95% CI: 1.091–1.150, *p* < 0.001; OR: 2.101, 95% CI: 1.308–3.372, *p* = 0.002; OR: 1.089, 95% CI: 1.013–1.171, *p* = 0.021).

**Figure 1:**
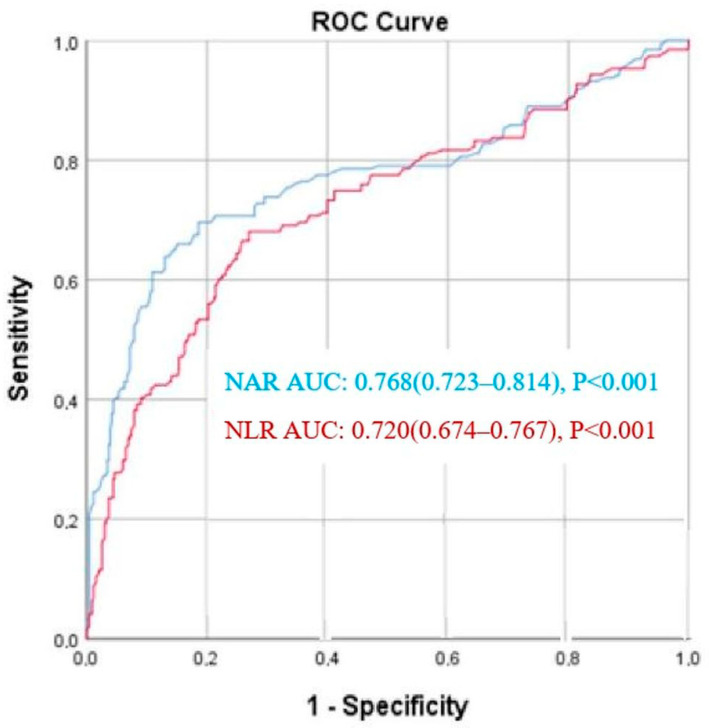
Receiver operating characteristic (ROC) curves for the Neutrophil/Albumin ratio (NAR), Neutrophil/Lymphocyte ratio (NLR) for predicting critical carotid artery stenosis.

In the ROC analysis, the best cut-off value for NAR to predict critical carotid artery stenosis was 1.47, with a sensitivity of 73.8% and specificity of 70.5% (area under the curve [AUC] = 0.768; 95% CI: 0.723–0.814; *p* < 0.001). The optimal cut-off value for NLR was 2.74, which predicted critical carotid artery stenosis with 70.7% sensitivity and 63% specificity; AUC was 0.720 (95% CI: 0.674–0.767; *p* < 0.001) (Figure 1).

## 4. Discussion

In our study, NAR values of patients with critical carotid artery stenosis were found to be significantly higher than those of patients without critical carotid artery stenosis. This finding suggests that NAR may be used as a potential biomarker in predicting critical carotid artery stenosis.

The atherosclerotic process is a highly complex pathophysiological event that develops in arterial walls and involves many cellular and molecular mechanisms. Since inflammatory cells such as neutrophils, lymphocytes, and platelets play an active role in almost every stage of atherosclerotic plaques, atherosclerosis is nowadays recognised as an inflammatory disease [27,28,29,30]. Despite all diagnostic and therapeutic advances, CVD, including carotid artery stenosis, remains a major public health problem on a global scale [28]. In carotid artery stenosis, a significant association between the degree of stenosis and stroke risk has been demonstrated. Therefore, considering the potential of interventional treatment modalities such as carotid endarterectomy or carotid artery stenting to reduce morbidity and mortality, accurate assessment of carotid artery stenosis severity is of great clinical importance [31,32,33,34].

The severity of carotid artery stenosis can be assessed using imaging modalities such as DUS, magnetic resonance angiography (MRA), DSA, and computed tomography angiography (CTA). However, due to the limited availability, relatively high cost, and side effects of these modalities, simpler, more affordable, and more readily available operator-independent clinical and laboratory markers are needed. In this context, considering the role of inflammation in atherosclerosis, simple laboratory markers based on inflammatory cell parameters (indicators such as NLR, PLR) may be potentially useful in the assessment of carotid artery stenosis severity. Indeed, inflammation markers such as NLR, PLR, and CRP have been used in the assessment of carotid artery stenosis severity and prognosis [35,36,37,38].

Recent studies have demonstrated that inflammation plays a critical role in the pathogenesis of carotid artery stenosis. The neutrophil-to-lymphocyte ratio (NLR) has emerged as a reliable indicator of stenosis severity, while the platelet-to-lymphocyte ratio (PLR) has been linked to both stenosis severity and increased stroke risk [14,27,35,39,40]. NLR has also shown prognostic value in identifying silent infarcts and predicting outcomes following endarterectomy [41,42]. The systemic immune–inflammation index (SII) has been identified as an independent predictor of stenosis severity, and C-reactive protein (CRP), as well as the CRP-to-albumin ratio (CAR), has also demonstrated correlations with disease severity [43,44]. Furthermore, inflammatory markers have been associated with restenosis development after treatment in patients with severe carotid artery stenosis [13]. Consistent with these findings, our study observed significantly higher NLR levels in patients with critical carotid artery stenosis, reinforcing the view that inflammatory markers reflect the aggressiveness or vulnerability of atherosclerotic disease rather than serving as direct measures of luminal narrowing. These markers may, therefore, be more valuable for early risk stratification and disease monitoring than for guiding intervention decisions on their own.

The neutrophil–albumin ratio (NAR) has attracted increasing attention as a powerful new biomarker reflecting the complex nature of inflammation. The NAR is calculated as the ratio of albumin, a negative acute phase reactant, to neutrophils, the active cell of inflammation, and is a more sensitive predictor of disease severity and prognosis than single parameter indicators [45,46,47]. Albumin is not only a protein but also a critical protector of vascular health with anti-inflammatory, antioxidant, anticoagulant, and antiplatelet aggregation properties. Low albumin levels accelerate the progression of carotid artery stenosis by predisposing to increased oxidative stress and foam cell formation in atherosclerotic plaques [48,49]. Neutrophils, as precursor cells of inflammation, initiate the basic mechanisms of vascular injury through pathological processes such as degranulation and neutrophil extracellular traps (NETs) that trigger tissue damage [50,51].

In this context, NAR stands out as a comprehensive index that measures both cellular activation and negative acute phase response of inflammation together and has a high diagnostic and prognostic value for carotid artery stenosis. NAR, which reflects the complex dynamics of systemic inflammation with a simple and accessible measurement, may have a high diagnostic and prognostic value for carotid artery stenosis.

The neutrophil–albumin ratio, although still a relatively new inflammatory marker, is of increasing importance in patients with carotid artery stenosis. Bao et al. [23] demonstrated that NAR is a strong predictor of poor functional outcomes 3 months after acute ischaemic stroke, which clearly highlights the role of inflammation in neurovascular injury. In their study by Zhang et al. [45,52,53], the significant association between NAR and prognosis after subarachnoid hemorrhage, as well as its correlation with acute stroke severity and neurological impairments by Mao et al. [24], indicates a direct link between the inflammatory burden of NAR with clinical outcomes. Shen et al. [25] reported the association between NAR and restenosis development in carotid artery stenosis patients and drew attention to the determinant role of inflammation in post-treatment complications. Our current study, in parallel with the literature, revealed that NAR was significantly elevated in severe carotid artery stenosis and supported that NAR may be a valuable biomarker in the diagnosis and prognosis of carotid artery stenosis as a comprehensive reflection of inflammation.

When evaluated together with composite inflammatory indices such as NLR, PLR, and CAR, NAR can be considered a valuable and reliable predictive parameter for carotid artery stenosis. Since such inflammatory markers are simple, rapid, easily measurable, and cost-effective indicators of inflammatory status, their integration into carotid artery stenosis risk models may facilitate early identification of high-risk patient groups. In our study, several inflammatory markers, including NAR, NLR, CRP, and CAR, were evaluated. Among these, NAR and NLR emerged as particularly valuable indicators of systemic inflammation related to carotid artery stenosis severity. Neutrophil-related ratios such as NAR and NLR not only reflect cellular activation but also the balance of inflammatory and anti-inflammatory responses, making them more sensitive and comprehensive markers compared to single parameters. Their simplicity, rapid measurability, and cost-effectiveness make them practical tools for integration into clinical risk models. By facilitating early identification of high-risk patients, these markers have the potential to guide timely interventions aimed at reducing ischemic stroke risk and improving outcomes in carotid artery stenosis. Future studies combining these biomarkers with advanced imaging techniques could further enhance diagnostic accuracy and prognostic assessment. Thus, targeted interventions to reduce the risk of ischaemic stroke and improve disease outcomes may become possible.

## 5. Conclusions

The findings of our study suggest that NAR can be used as a meaningful biomarker to predict the severity of carotid artery stenosis. NAR has the potential to be a practical tool for risk stratification, as it can measure the systemic manifestation of inflammation in a simple and accessible way. However, large-scale, multicentre, and prospective studies involving different patient populations are needed to integrate these results into clinical use. Furthermore, further testing of the predictive power of the NAR in randomized and controlled clinical trials will reinforce the clinical value of this biomarker. Evaluation of NAR, together with other inflammatory markers (such as NLR, CRP, and CAR) and advanced imaging methods (CT angiography and MRI), may allow a more comprehensive and sensitive analysis of carotid artery stenosis. Therefore, within the framework of multiparametric approaches, we believe that NAR can be evaluated not only as an independent biomarker but also as a parameter that can complement other clinical and imaging methods and contribute to diagnostic evaluation processes.

### Limitations

This study’s design is single-center and retrospective, and it included a limited number of patients. This weakens the ability of our results to apply to the broader population. More comprehensive, prospective, multicenter, and randomized controlled trials are needed to support our findings. We lacked baseline information on plaque morphology/vulnerability in carotid artery stenosis, which is a significant limitation. Furthermore, while a high NAR has been suggested to be effective in predicting the severity of carotid artery stenosis, we cannot make any statements regarding its impact on therapeutic management. The relatively low prevalence of hypercholesterolemia observed in the included patients (12.4%) may reflect missing or undocumented diagnoses in the retrospective data. Pharmacological data on anti-inflammatory treatments, including duration of treatment and patient compliance, were lacking, limiting our ability to assess their effects on inflammatory parameters.

## Figures and Tables

**Table 1:** Demographic characteristics of the study populations.

	Carotid Artery Stenosis		
	Non-Critical	Critical	*p* Value
Variables	(*n* = 434)	(*n* = 191)	-
Previous TIA/stroke	139 (32%)	60 (31.4%)	0.126
Previous CAD	54 (12.4%)	18 (9.4%)	0.087
Previous PAD	17 (3.9%)	10 (5.2%)	0.654
Age (years)	51 (45–57)	60 (54–68)	<0.001
Male sex (*n*, %)	329 (75.8%)	137 (71.7%)	0.260
Diabetes Mellitus (*n*, %)	143 (32.9%)	83 (43.5%)	0.025
Hypertension (*n*, %)	193 (44.5%)	104 (54.5%)	0.021
Hypercholesterolemia (*n*, %)	60 (13.8%)	38 (19.9%)	0.055
Current smoker (*n*, %)	139 (32%)	71 (37.2%)	0.063
BMI (kg/m^2^)	27.9 + 4.6	28.3 + 4.7	0.241
LVEF (%)	57.3 + 10	55.8 + 11	0.131

Abbreviations: TIA: Transient ischemic attack, CAD: Coronary artery disease, PAD: Peripheral artery disease, BMI: Body mass index, LVEF: Left ventricular ejection fraction.

**Table 2:** Laboratory findings of the study populations.

	Carotid Artery Stenosis		
	Non-Critical	Critical	*p* Value
Number of patients	(*n* = 434)	(*n* = 191)	-
Hemoglobin (mg/dL)	14.3 + 4.4	14.6 + 6.1	0.654
Platelets (10^3^/µL)	226.8 + 58	227.2 + 63	0.891
WBC (10^3^/µL)	7.7 + 3.2	12.6 + 5.9	<0.001
Neutrophil (10^3^/µL)	4.3 (3.2–6.2)	8.1 (4.9–12.5)	<0.001
Lymphocyte (10^3^/µL)	1.8 (1.3–2.5)	1.7 (1.3–2.4)	0.261
NLR	2.2 (1.4–3.2)	4.2 (2.3–8.9)	<0.001
NAR	1.1 (0.8–1.6)	2.1 (1.4–3.2)	<0.001
CRP	3.8 (1.8–8)	5.7 (3.6–7.6)	0.005
CAR	0.9 (0.5–1.9)	1.6 (0.8–2.1)	<0.001
Glucose (mg/dL)	125 (104–173)	128 (105–173)	0.409
Creatinine (mg/dL)			
AST (U/L)	19.0 + 7.4	19.3 + 4.7	0.703
ALT (U/L)	21.2 + 11	23.9 + 14	0.102
Albumin (mg/dL)	3.8 (3.5–4.2)	3.6 (3.5–4.2)	0.294
Total cholesterol (mg/dL)	176.7 + 46	171.9 + 52	0.296
High density lipoprotein cholesterol (mg/dL)	37.2 + 13	37.4 + 10	0.946
Low density lipoprotein cholesterol (mg/dL)	114.4 + 42	113 + 45	0.736
Triglyceride (mg/dL)	129 + 31	143 + 36	0.280

Abbreviations: WBC: White blood cell, NLR: Neutrophil/Lymphocyte ratio, NAR: Neutrophil/Albumin ratio, CRP: High sensitive C-reactive protein, CAR: CRP/Albumin ratio, ALT: Alanine aminotransferase, AST: Aspartate aminotransferase.

**Table 3:** Univariate and multivariate of predictors of critical carotid artery stenosis.

	Univariate Analysis			Multivariate Analysis		
	Odds Ratio	95% CI	*p* Value	Odds Ratio	95% CI	*p* Value
Age	1.102	1.081–1.124	<0.001	1.120	1.091–1.150	<0.001
Hypertension	1.498	1.063–2.110	0.021	2.101	1.308–3.372	0.002
Diabetes Mellitus	1.489	1.050–2.123	0.025			
WBC ^a^	1.308	1.239–1.381	<0.001			
NAR ^a^	3.197	2.542–4.022	<0.001	3.432	2.116–5.566	<0.001
NLR ^a^	1.253	1.186–1.323	<0.001	1.089	1.013–1.171	0.021
CRP ^b^	1.045	1.003–1.089	0.034			
CAR ^b^	1.315	1.111–1.557	0.001			

CI: Confidence interval. Abbreviations: WBC: White blood cell, NAR: Neutrophil/Albumin ratio, CAR: CRP/Albumin ratio, NLR: Neutrophil/Lymphocyte ratio, CRP: High sensitive C-reactive protein. ^a,b^: These parameters were not entered into the model to prevent multicollinearity.
